# Survival is influenced by approaches to local treatment of Ewing sarcoma within an international randomised controlled trial: analysis of EICESS-92

**DOI:** 10.1186/s13569-018-0093-y

**Published:** 2018-03-30

**Authors:** Jeremy Whelan, Allan Hackshaw, Anne McTiernan, Robert Grimer, David Spooner, Jessica Bate, Andreas Ranft, Michael Paulussen, Herbert Juergens, Alan Craft, Ian Lewis

**Affiliations:** 1 Department of Oncology, University College Hospitals London NHS Foundation Trust, 250 Euston Road, London, NW1 2PG UK; 2 Cancer Research UK and UCL Clinical Trials Centre, University College London, London, UK; 3 Children’s Cancer and Leukaemia Group Data Centre, Cancer Studies and Molecular Medicine, University of Leicester, Leicester, UK; 4 The Royal Orthopaedic Hospital, Birmingham, UK; 5 Queen Elizabeth II Hospital, Birmingham, UK; 6 University Hospital Essen, Essen, Germany; 7 Vestische Kinder- und Jugendklinik Datteln, University Witten/Herdecke, Datteln, Germany; 8 Department of Pediatric Hematology and Oncology, University Children’s Hospital Münster, Münster, Germany; 9 Northern Institute for Cancer Research, Newcastle University, Newcastle upon Tyne, UK; 10 University of Leeds and Leeds Community Healthcare Trust, Leeds, UK

**Keywords:** Ewing sarcoma, Local therapy

## Abstract

**Background:**

Two national clinical trial groups, United Kingdom Children’s Cancer and Leukaemia Group (CCLG) and the German Paediatric Oncology and Haematology Group (GPOH) together undertook a randomised trial, EICESS-92, which addressed chemotherapy options for Ewing’s sarcoma. We sought the causes of unexpected survival differences between the study groups.

**Methods:**

647 patients were randomised. Cox regression analyses were used to compare event-free survival (EFS) and overall survival (OS) between the two study groups.

**Results:**

5-year EFS rates were 43% (95% CI 36–50%) and 57% (95% CI 52–62) in the CCLG and GPOH patients, respectively; corresponding 5-year OS rates were 52% (95% CI 45–59%) and 66% (95% CI 61–71). CCLG patients were less likely to have both surgery and radiotherapy (18 vs. 59%), and more likely to have a single local therapy modality compared to the GPOH patients (72 vs. 35%). Forty-five percent of GPOH patients had pre-operative radiotherapy compared to 3% of CCLG patients. In the CCLG group local recurrence (either with or without metastases) was the first event in 22% of patients compared with 7% in the GPOH group. After allowing for the effects of age, metastases, primary site, histology and local treatment modality, the risk of an EFS event was 44% greater in the CCLG cohort (95% CI 10–89%, p = 0.009), and the risk of dying was 30% greater, but not statistically significant (95% CI 3–74%, p = 0.08).

**Conclusions:**

Unexpected differences in EFS and OS occurred between two patient cohorts recruited within an international randomised trial. Failure to select or deliver appropriate local treatment modalities for Ewing’s sarcoma may compromise chances of cure.

*Trial registration* Supported by Deutsche Krebshilfe (Grants No. DKH M43/92/Jü2 and DKH 70-2551 Jü3), and European Union Biomedicine and Health Programme (Grants No. BMH1-CT92-1341 and BMH4-983956), and Cancer Research United Kingdom. Clinical trial information can be found for the following: NCT0000251

## Background

Collaboration between national clinical study groups to run large randomised trials is advantageous, especially in rare disease settings. It allows rapid accrual of larger numbers of patients to provide sufficient power for robust analyses. Indeed, joint studies may be the only means of effectively answering randomised questions in rare cancers [1, 2]. EICESS-92, a trial developed and completed by the Children’s Cancer and Leukaemia Group (CCLG, formerly United Kingdom Children’s Cancer Study Group, UKCCSG) and the Cooperative Ewing’s Sarcoma Studies (CESS) group of the German Paediatric Oncology and Haematology Group (GPOH) with associated institutions in Austria, Switzerland and the Netherlands, addressed two chemotherapy questions in patients with Ewing sarcoma (ES). It remains one of the largest randomised studies conducted in this cancer.

The primary aims of the trial were to demonstrate an increase in event-free survival (EFS), and decreased treatment-related morbidity for patients with standard risk disease. The overall results of the trial have been reported [3]. However, we found evidence that EFS and overall survival (OS) differed between the CCLG and GPOH study groups. Although these data have been reported in abstract form, this more detailed analysis retains relevance and influence on practice [4].

## Patients and methods

**Fig. 1:**
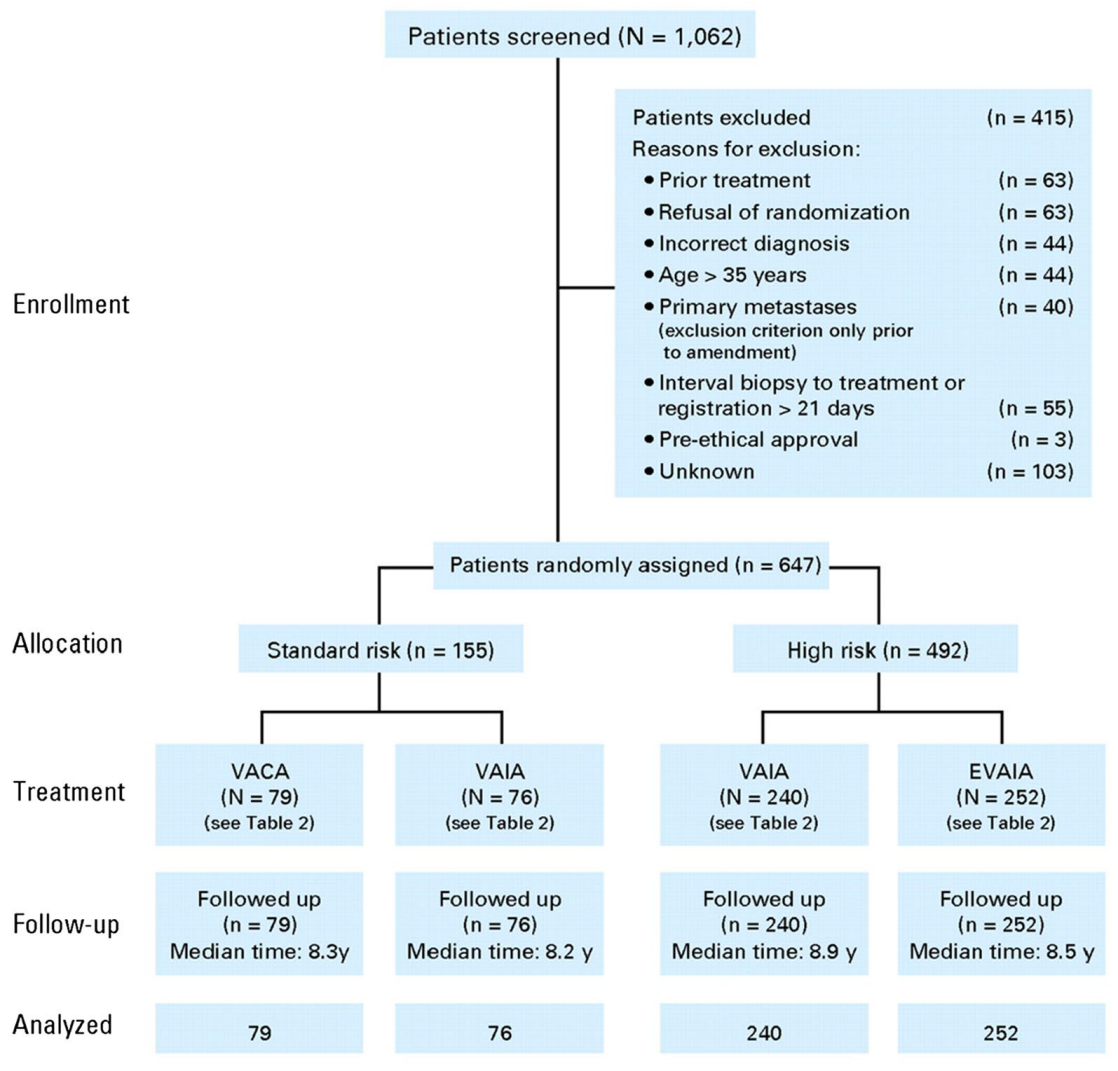
EICESS 92 consort diagram (from original publication Paulussen et al. [3])

The trial design of EICESS-92 is outlined in Fig. 1, and details are described elsewhere [3]. The study was confined to patients with primary tumours of bone.

Patients with localised tumours of < 100 ml were classified as standard risk (SR), patients with large localised tumours (≥ 100 ml), or with metastatic disease, were classified as high risk (HR). Patients were randomly assigned to one of two treatment arms. SR-patients received four VAIA courses (vincristine, doxorubicin, ifosfamide, actinomycin D) followed by ten courses of either VAIA or VACA (cyclophosphamide instead of ifosfamide) whilst HR-patients were randomised to either fourteen courses of VAIA or fourteen courses of VAIA with etoposide (EVAIA) [3].

Surgery and/or radiotherapy to the primary tumour (‘local therapy’) were scheduled to occur after four cycles of chemotherapy, at week 12. The choice of local therapy was made by clinicians for individual patients. The protocol was permissive but indicated that surgery should be undertaken whenever possible. Preoperative radiotherapy (44.8 Gy) was recommended when there was < 50% reduction of a soft tissue component, evident on repeat imaging after 2 chemotherapy courses. Radiotherapy (54.4 Gy) replaced surgery for tumours deemed inoperable. Post-operative radiotherapy (54.4 Gy) was recommended after intralesional surgery or marginal surgery with poor response (< 90% necrosis). Postoperative radiotherapy (44.8 Gy) was to be considered for marginal resections with good response (≥ 90% necrosis) or wide resections with poor response. Hyperfractionated irradiation was recommended in these cases.

In the CESS group, the practice for treating clinical teams of routinely seeking advice from clinicians in the central trials office is well established including detailed guidance about radiotherapy planning [5]. No similar process was in place in the UK although the majority of patients selected for surgery by local clinicians were operated on at four centres only.

### Statistical methods

The EICESS database was frozen in March 2007. EFS was calculated from the date of randomisation until the date of relapse, death or second malignancy, whichever occurred first. OS was calculated from the date of randomisation until date of death. Patients alive at last follow-up were censored at date last seen. Kaplan–Meier survival curves were examined, and Cox regression modelling was used to investigate differences after adjusting for multiple factors, producing hazard ratios (HR). Results are also presented separately for patients with only localised disease.

## Results

Between 1992 and 1998, 647 patients were randomised: 210 CCLG and 437 GPOH (CONSORT diagram Fig. 1). The median follow-up was 8.5 years.

### Patient characteristics

These were largely similar between the trial groups, except that CCLG patients tended to have more extremity tumours (Table 1; 55 vs. 45%), fewer central tumours (40 vs. 54%), and fewer with atypical Ewing’s sarcoma (4 vs. 16%), compared to GPOH patients.

**Table 1:** Comparison of patient characteristics between CCLG and GPOH

	Number of patients (percentage)		p value
	CCLG	GPOH	
	N = 210	N = 437	
Gender			
Female	84 (40)	177 (40)	0.90
Male	126 (60)	260 (60)	
Age (approx quartiles) (years)			
0–9	34 (16)	94 (22)	0.17
10–14	74 (35)	123 (28)	
15–19	58 (28)	115 (26)	
20–35	44 (21)	105 (24)	
Primary site			
Central axis	84 (40)	236 (54)	< 0.001 (0.004)
Extremity	115 (55)	197 (45)	
Unknown	11 (5)	4 (1)	
Axial skeletal	26 (12)	89 (20)	
Spine	7 (3	30 (7)	
Pelvis	51 (24)	117 (27)	
Limb proximal	61 (29)	103 (23)	
Limb distal	54 (26)	94 (22)	
Unknown	11 (5)	4 (1)	
Volume			
< 100 ml	57 (27)	117 (27)	0.48 (0.97)
≥ 100 ml	149 (71)	304 (69)	
Unknown	4 (2)	16 (4)	
Metastases			
No	150 (71)	329 (75)	0.27 (0.41)
Yes^d^	56 (27)	105 (24)	
Unknown	4 (2)	3 (1)	
Histology			
Ewing’s sarcoma	140 (67)	261 (60)	< 0.001 (< 0.001)
Atypical Ewing’s	8 (4)	70 (16)	
PNET	43 (20)	101 (23)	
Other^a^	6 (3)	5 (1)	
Unknown	13 (6)	0 (0)	
Risk group			
Standard (SR)	53 (25)	102 (23)	0.60
High (HR)	157 (75)	335 (77)	
Trial treatment			
SR-VACA	27 (13)	52 (12)	0.96
SR-VAIA	26 (12)	50 (11)	
HR-VAIA	76 (36)	164 (38)	
HR-EVAIA	81 (39)	171 (39)	
Histological response^c^			
Good	52 (25) [58]	78 (18) [65]	< 0.001 [0.33]
Poor	37 (18) [42]	42 (10) [35]	
No surgery	103 (49)	111 (25)	
NA^b^	3 (1)	200 (46)	
Unknown	15 (7)	6 (1)	
No. of chemotherapy cycles received			
1–4	18 (8.6)	26 (6.0)	0.32
5–9	22 (10.5)	62 (14.2)	
10–13	36 (17.10	83 (19.0)	
14	131 (62.4)	254 (58.2)	
Unknown	3 (1.4)	12 (2.8)	

p values including unknown data; the p values in brackets exclude unknown data

^a^Osteosarcoma or soft tissue

^b^NA not applicable, i.e. patients with early radiotherapy before surgery

^c^The numbers in square brackets are based only on patients with a good or poor response

^d^The proportions of patients with bone or bone marrow metastases were similar: 8% GPOH and 5% CCLG

Where histological response data were available for patients who did not receive pre-operative radiotherapy, there was no significant difference in the proportion of patients from the two study groups with either a good or poor histological response (Table 1, p = 0.33).

## Chemotherapy and local therapy

There was no evidence of a difference in the delivery of chemotherapy between groups. The number of chemotherapy cycles received (Table 1) and the median total dose for each cytotoxic drug administered per patient were similar. A similar proportion completed all 14 cycles; 62% vs. 58% in the CCLG and GPOH groups, respectively (p = 0.30).

Table 2 shows the type of local therapy used in CCLG and GPOH patients. Most CCLG patients (72%) had a single therapy (surgery alone or radiotherapy alone); whilst most GPOH patients had both radiotherapy and surgery (59%), which was mainly radiotherapy followed by surgery (45%). Only 18% of CCLG patients had both radiotherapy and surgery. A similar pattern was seen for patients without metastatic disease.

**Table 2:** Comparison of local treatment modality in CCLG and GPOH

	Number of patients (percentage)		p value
	CCLG	GPOH	
	N = 210	N = 437	
Local treatment modality			
Surgery alone	70 (33)	71 (16)	< 0.001^a^
Radiotherapy alone	81 (39)	85 (19)	
Radiotherapy then surgery	6 (3)	195 (45)	
Surgery then radiotherapy	32 (15)	60 (14)	
None (progressive disease)	18 (9)	7 (2)	
Unknown	3 (1)	19 (4)	
Localised disease only			
Surgery alone	59 (39)	63 (19)	< 0.001
Radiotherapy alone	53 (35)	55 (17)	
Radiotherapy then surgery	5 (3)	147 (45)	
Surgery then radiotherapy	24 (16)	47 (14)	
None (progressive disease)	9 (6)	3 (1)	
Unknown	0	14 (4)	

^a^p value for the association between type of local treatment and study group. The p value is also < 0.001 if ‘none’ or ‘unknown’ are excluded

Patient characteristics were examined which might influence the selection of local treatment (Table 3). Many patients with metastatic disease were treated with radiotherapy alone in both trial groups, though the percentage was higher in CCLG patients. A substantial proportion of CCLG patients with central axis tumours had radiotherapy alone (62%), while those with extremity tumours tended to have surgery alone. CCLG patients were more likely to have both radiotherapy and surgery if they had extremity tumours compared to central axis tumours (23 vs. 11%). A similar pattern was seen in GPOH patients, though there was much less of a difference in the proportion who had both therapies depending on whether they had extremity or central axis tumours (64% vs. 54%). While there was no evidence of an association between choice of local treatment and either tumour volume (p = 0.44) or age (p = 0.12) in GPOH patients, there was evidence of this in CCLG patients. Those with a volume < 100 ml were more likely to have surgery alone (47%), and those with a volume ≥100 ml tended to have radiotherapy alone. Proportionally more patients with volume ≥100 ml received both therapies compared to those with volume < 100 ml (21 vs. 10%). In the CCLG group there was a clear trend with age; the proportions receiving single modality treatment were 94% (age 0–9 years), 75% (age 10–14 years), 68% (age 15–19 years) and 59% (age 20–35 years), indicating that older patients tended to be given both therapies.

**Table 3:** Association between the choice of local modality treatment and specified patient characteristics

	Number of patients (percentage), excluding missing data									
	CCLG					GPOH				
	N	None	RT alone	Surgery alone	RT and surgery	N	None	RT alone	Surgery alone	RT and surgery
Disease										
Localised	150	9 (6)	53 (35)	59 (39)	29 (19)	315	3 (1)	55 (17)	63 (20)	194 (62)
Metastatic	56	9 (16)	27 (48)	11 (20)	9 (16)	101	4 (4)	30 (30)	8 (8)	59 (58)
Localised extremity disease	91	5 (5)	18 (20)	46 (50)	22 (24)	155	1 (1)	8 (5)	44 (28)	102 (66)
Localised pelvic disease	31	0	24 (77)	6 (19)	1 (3)	79	1 (1)	27 (36)	7 (9)	39 (53)
Primary site										
Central axis	84	9 (11)	52 (62)	14 (17)	9 (11)	226	4 (2)	73 (32)	22 (10)	127 (56)
Extremity	115	7 (6)	28 (24)	54 (47)	26 (23)	189	3 (2)	11 (6)	48 (25)	127 (67)
Volume (ml)										
< 100	57	5 (9)	19 (33)	27 (47)	6 (10)	111	3 (3)	26 (23)	16 (14)	66 (59)
≥ 100	146	13 (9)	61 (42)	40 (27)	32 (22)	292	4 (1)	54 (18)	52 (18)	182 (62)
Age (years)										
0–9	34	0	10 (29)	22 (65)	2 (6)	92	1 (1)	18 (20)	22 (24)	51 (55)
10–14	74	5 (7)	32 (43)	24 (32)	13 (18)	118	0	30 (25)	11 (9)	77 (65)
15–19	56	5 (9)	25 (45)	13 (23)	13 (23)	111	3 (3)	21 (19)	21 (19)	66 (59)
20–35	43	8 (19)	14 (33)	11 (26)	10 (23)	97	3 (3)	16 (16)	17 (18)	61 (63)

Chi square tests were used to examine the association between each factor and choice of local therapy, excluding those who received no local treatment (‘None’ in the table)

CCLG: disease (p = 0.04); primary site (p < 0.001); volume (p = 0.01); age (p = 0.01)

GPOH: disease (p = 0.002); primary site (p < 0.001); volume (p = 0.44); age (p = 0.12)

*N* total number of patients *RT* radiotherapy

### Overall outcome

**Fig. 2:**
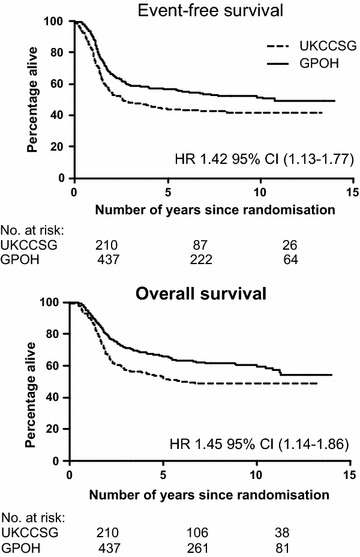
Event-free and overall survival for CCLG and GPOH patients. 5-year EFS rates: CCLG 43% (95% CI, 36–50%); GPOH 57% (95% CI 52–62). 5-year OS rates: CCLG 52% (95% CI 45–59%); GPOH 66% (95% CI 61–71). 10-year EFS rates: CCLG 41% (95% CI 35–48); GPOH 51% (95% CI 46–56). 10-year OS rates: CCLG 49% (95% CI 42–56); GPOH 60% (95% CI 55–65)

Appendix Table 6 shows the distribution of events and deaths by trial group. The CCLG cohort had more local relapses (with or without metastatic disease) than GPOH; 22 vs. 7%. Appendix Tables 7, 8 show the distribution of events according to local therapy. Figure 2 shows EFS and OS according to trial group. Both outcomes were poorer in the CCLG patients.

### Differences in survival between the trial groups allowing for specified factors

The risk of having an event or dying for CCLG patients compared to GPOH was examined using Cox regression modelling. Overall, the chance of having an event (relapse, death or second malignancy) was increased by 42% (HR 1.42, 95% CI 1.13–1.77, p = 0.002) in the CCLG group compared to the GPOH group. The CCLG group had an increased risk of dying of 45% (HR 1.45, 95% CI 1.14–1.86, p = 0.003) in comparison to the GPOH group (Appendix Table 9). The table also shows that the excess risk (42% EFS, and 45% OS) did not materially change, even after allowing for several prognostic factors: age, metastatic disease status, primary site or histology. The association between outcome and study group was very similar when only examining patients with non-metastatic disease. Combined local treatment seemed to have an effect on OS (reducing the excess risk from 45 to 30%) but not EFS. When several prognostic factors were allowed for together, there was still an increased risk among CCLG patients: 44% for EFS (HR 1.44, p = 0.009) and 30% for OS (HR 1.30, p = 0.08, which was not statistically significant). We further examined the effect separately among patients with localised disease only and those with metastatic disease. The EFS and OS hazard ratios were: 1.47 (95% CI 1.11–1.96) and 1.52 (95% CI 1.11–2.07) for those with localised disease only. For those with metastatic disease, the HRs for EFS and OS were: 1.20 (95% CI 0.82–1.74) and 1.22 (95% CI 0.82–1.80) based on all patients, and 0.98 (95% CI 0.65–1.48) and 1.01 (0.66–1.57) based on those who had local therapies.

In an analysis in which only patients that had a local recurrence (with or without distant recurrence) were counted as an event (all other events censored at the time when they occurred), an excess risk was still found in CCLG patients compared to GPOH. The hazard ratios were: 3.46 (95% CI 2.19–5.47) unadjusted, and 3.47 (95% CI 2.00–6.01), allowing for age, primary site, histology and local treatment.

Appendix Table 9 also shows hazard ratios for CCLG compared to GPOH patients only among those who received local treatment. The HRs were 1.22 and 1.28 for EFS and OS, respectively. These estimates were somewhat lower than those in all patients (and only just missed statistical significance, due to being based on a smaller number of patients), indicating that the difference between the HRs based on all patients and those based only on those who had local treatment is largely due to excluding those with progressive disease or missing data on local therapy. The EFS HR of 1.22 reduced to 1.14 after allowing for the type of local treatment, i.e. it is partly explained by differences in the local therapy administered (consistent with Table 2) further indicating the influence of local treatments on survival outcomes.

When we examined the effect of different local treatment modalities on outcomes, there was no evidence of a difference between CCLG and GPOH patients for either EFS or OS among those who received radiotherapy alone (Table 4). This is not surprising given that the proportions with a local relapse (with or without metastases) were not very different: 22% CCLG vs. 16% GPOH (Appendix Table 7, 8). The risk of an event or death was moderately higher in the CCLG group among patients who had surgery alone (excess risk: EFS 31% and OS 50%, though neither were statistically significant). However, among patients who had both radiotherapy and surgery (Table 4), CCLG patients were 67% more likely to have an event (p = 0.03) and 65% more likely to die (p = 0.05). The adjusted point estimates were similar but were not statistically significant (p = 0.07 for both EFS and OS). To further consider different numbers of CCLG and GPOH patients who received radiotherapy then surgery or vice versa, Table 4 shows HRs within in each subgroup: there was still evidence of an excess risk among CCLG patients. Appendix Table 10 is only based on patients without metastatic disease: the conclusions were similar to Table 4. Appendix Table 11 is based only on patients with metastatic disease: it is difficult to make any reliable conclusions because of the smaller patient numbers.

**Table 4:** Hazard ratios (CCLG vs. GPOH) according to local treatment modality

	Local treatment modality				Subdivision of RT and surgery group	
	None N = 23	Radiotherapy (RT) alone N = 164	Surgery alone N = 138	RT and surgery N = 289	RT then surgery N = 201	Surgery then RT N = 88
No. events						
EFS	21	105	53	131	93	38
OS	19	92	41	109	79	30
Unadjusted						
EFS	20 (2.64–161)	0.86 (0.58–1.26)	1.31 (0.76–2.25)	1.67 (1.05–2.66)	2.22 (0.81–6.07)	1.99 (1.05–3.78)
OS	1.50 (0.56–3.96)	0.95 (0.63–1.44)	1.50 (0.81–2.80)	1.65 (1.00–2.74)	1.96 (0.72–5.37)	2.10 (1.02–4.30)
Adjusted for age, metastatic disease, primary site and histology						
EFS	53 (4.0–477)	0.92 (0.62–1.38)	1.24 (0.70–2.20)	1.82 (1.12–2.94)	2.40 (0.84–6.84)	2.50 (1.24–5.06)
OS	2.09 (0.64–6.79)	1.06 (0.69–1.63)	1.41 (0.73–2.74)	1.81 (1.07–3.05)	2.21 (0.77–6.35)	2.76 (1.26–6.05)
Adjusted for age, metastatic disease, primary site, histology and time between the start of chemotherapy and starting local treatment						
EFS		0.91 (0.60–1.38)	1.24 (0.70–2.19)	1.61 (0.96–2.70)	1.98 (0.70–5.60)	2.83 (1.30–6.16)
OS		1.04 (0.67–1.63)	1.43 (0.73–2.78)	1.68 (0.97–2.91)	1.94 (0.68–5.58)	3.39 (1.42–8.06)

Hazard ratios greater than 1 indicate that CCLG patients had a higher risk of having an event or dying compared to GPOH patients

Based on data excluding patients with unknown primary site because there were so few

*EFS* event-free survival; *OS* overall survival

### Local treatment and timing of treatment

GPOH patients were more likely to have “early” local therapy, i.e. within 12 weeks of starting chemotherapy, compared to CCLG patients: 43% (176/407) vs. 9% (17/180), p < 0.001. This is consistent with the greater use of pre-operative radiotherapy. GPOH patients were also less likely to have “late” local therapy, i.e. more than 15 weeks from start of chemotherapy; 20% (82/407), vs. 32% (57/180) p = 0.004. There was an association between clinical outcome and the length of time from the start of chemotherapy to the start of local therapy (considered as a continuous variable). For every increase of 4 weeks, the risk of an (EFS) event increased by 27% (HR 1.27, 95% CI 1.05–1.53) among patients who had pre-operative radiotherapy; 14% (HR 1.14, 95% CI 1.02–1.27) among those who had surgery, with or without subsequent radiotherapy; and 7% (HR 1.07, 95% CI 0.96–1.19) among those who had radiotherapy alone.

Appendix Table 12 examines the influence of type of local treatment and its timing (in all patients and only those with localised disease). Either factor reduced the HRs for EFS and OS to a similar extent. In the multivariate model they were each independent prognostic factors. However, Table 4 and Appendix Table 10 show that when the data were presented by type of treatment, the timing had some effect but it still did not largely explain the difference between CCLG and GPOH outcomes (surgery with or without radiotherapy).

### Localised extremity tumours

Table 5 shows the hazard ratios comparing CCLG with GPOH, according to primary site. When 253 patients with localised extremity tumours were examined, statistically significant survival differences remained between the two study groups. There was a 68% increase in the death rate among CCLG patients compared to those in GPOH, after allowing for local therapy and other factors (Table 5, p = 0.05). The 5-year survival rates were: GPOH 81% (95% CI 75–87%), and CCLG 62% (95% CI 52–72%). There were no differences in the baseline patient characteristics or number of chemotherapy cycles received, except a slight excess of atypical ES in GPOH; 15% (24/162) vs. 5% (5/91) in CCLG. For patients with localised extremity tumours, combined modality treatment was used more frequently in GPOH patients than CCLG patients (66% vs. 24%) whereas a greater proportion of CCLG patients were treated with radiotherapy alone (20% vs. 5%). More CCLG patients had a local recurrence, with or without metastatic disease (16% vs. 3%).

**Table 5:** Hazard ratios (CCLG vs. GPOH) according to primary site and localised disease

	No. events	Unadjusted		Adjusted for age, metastatic disease, histology and local treatment	
		Hazard ratio 95% CI	p value	Hazard ratio 95% CI	p value
Central axis					
All (n = 320)					
EFS	177	1.48 (1.08–2.03)	0.02	1.27 (0.89–1.82)	0.19
OS	154	1.47 (1.05–2.06)	0.03	1.20 (0.82–1.75)	0.36
Localised (n = 220)					
EFS	102	1.47 (0.97–2.24)	0.07	1.33 (0.82–2.16)	0.24
OS	87	1.46 (0.93–2.30)	0.10	1.15 (0.69–1.92)	0.58
Extremity					
All (n = 312)					
EFS	140	1.56 (1.12–2.18)	0.009	1.75 (1.18–2.60)	0.005
OS	112	1.69 (1.17–2.45)	0.006	1.59 (1.02–2.46)	0.04
Localised (n = 253)					
EFS	99	1.68 (1.13–2.50)	0.01	1.56 (1.00–2.43)	0.05
OS	75	1.86 (1.18–2.93)	0.007	1.68 (1.00–2.81)	0.05
Pelvic disease					
All (n = 168)					
EFS	100	1.36 (0.90–2.05)	0.14	1.05 (0.65–1.70)	0.84
OS	90	1.32 (0.85–2.04)	0.21	0.98 (0.60–1.62)	0.94
Localised (n = 107)					
EFS	56	1.22 (0.69–2.15)	0.51	0.98 (0.51–1.89)	0.96
OS	51	1.17 (0.64–2.14)	0.61	1.01 (0.51–2.03)	0.97

Hazard ratios greater than 1 indicate that CCLG patients had a higher risk of having an event or dying compared to GPOH patients

Hazard ratios for localised disease were not adjusted for metastatic disease

*EFS* event-free survival; *OS* overall survival

### Central axis and pelvic tumours

Among patients with central axis tumours, the HRs for both EFS and OS reduced after allowing for several factors, and most of the reduction was due to adjusting for local treatment, indicating that this does have a role. A more pronounced reduction was seen for patients with pelvic disease (HRs: EFS 1.05, OS 0.98). Patients with localised pelvic tumours had a similar survival whether treated in the CCLG or GPOH: the 5-year OS rates were 52 and 56%, respectively (p = 0.65), and the adjusted OS HR was 1.01, 95% CI 0.51–2.03 (Table 5), allowing for the different local treatment modalities used between the two cohorts. Radiotherapy alone was the local treatment modality used in 77% (24/31) CCLG patients compared to 34% (27/79) GPOH patients. Surgery combined with radiotherapy was only used for 3% of CCLG patients (1/31) compared to 49% of GPOH patients (39/79). A survival advantage seemed evident for patients with localised pelvic tumours selected for surgery, compared to those who had radiotherapy alone (hazard ratio 0.50, 95% CI 0.28–0.88, p = 0.016).

## Discussion

The EICESS-92 clinical trial revealed unexpected differences in survival between cohorts of ES patients from two countries. Differences in mortality from cancer between countries are well documented in Europe, especially for common cancers [6, 7]. These differences in outcome have also been reported for rare cancers [8, 9]. Survival in the UK is lower for some cancers than in other Western European and Nordic countries. Explanations for these differences may include: registry data being unrepresentative or containing artefact; differences in population health or use of health resources; differences in stage of cancer at diagnosis and variable access to optimal treatment or expertise [10]. Within EUROCARE 3, which examined registry data for 20 European countries, 5-year survival from ES ranged from 31 to 86% for the period 1990–1994 [11]. The EUROCARE-5 study investigated whether survival differences among European countries had changed further from 1999 to 2007 and found persisting inequalities both for children and adolescents and young adults [12, 13]. The main influences on continued survival disparity are attributable to lack of health-care resources and access to modern treatments, lack of specialised centres with multidisciplinary teams, delayed diagnosis and treatment and poor management of treatment, and drug toxicity. However, this is unlikely to fully account for the wide range in survival from ES reported here.

Given that all patients were treated according to a common protocol, the substantial survival differences between national study groups in this randomised trial are striking. Survival for the entire group of 647 patients exceeded 60% but this disguises the 14% inferior 5 year survival of the cohort of patients recruited through the CCLG. The inferior outcome was not obviously accounted for by differences in baseline characteristics, delivery of chemotherapy or follow up. Differences were found in management of the primary tumour and in the rates of local recurrence associated with different treatment modalities. We believe that this evidence provides support that variations in local therapy influence survival.

It is possible that inherent differences in health care delivery systems between the two study groups may have contributed to survival differences. No differences were found in the tumour volume and the frequency of presentation with metastases between the two study groups, factors which might indicate systematic delays in diagnosis in one study group compared to the other. Likewise, there was no indication of a systematic difference in the way chemotherapy was delivered.

Approaches to local tumour control were clearly different between the two groups, including the timing of local treatments, but they did not explain all of the difference, particularly when patients had surgery. Primary tumour control in ES can be achieved with surgery, radiotherapy or a combination of both. The choice is based on balancing the differing morbidities of the two modalities for each individual patient. The optimal approach for local control remains a topic of debate. The relative merits of surgery and radiotherapy have been debated but conclusions are often obscured by patient selection which biases comparison [5, 14–17]. Tumours that are inoperable and thus treated by radiotherapy alone are often associated with other adverse features such as large volume [18–21]. The greater incidence of local relapse in CCLG patients indicates that both selection of patients for, and delivery of, surgery and radiotherapy may have been sub-optimal.

While not specific to Ewing sarcoma, there is a general consensus on the relevance of centralization to high volume centres and networks for sarcoma, especially for diagnosis and surgery [22, 23]. The degree of centralisation and the process of decision-making about local therapy differed between the two study groups in EICESS-92. Ideally, the optimal local treatment for an individual patient should be decided through consideration of patient characteristics, the potential benefit and harm of the treatment options, and patient preference. In the CESS group, treatment took place in three hundred or more centres, most of which treat relatively few patients. However, each centre was familiar with accessing specialist guidance from the trial headquarters. This extended to a centralised system of advice for local therapy planning [5]. A consequence is likely to have been considerable consistency of local treatment approach within the majority of the GPOH cohort. In contrast, although surgery for bone sarcomas took place mainly in four centres in the UK, advice about local tumour management was only sought on an ad hoc basis and there was no similar system for any degree of central treatment planning.

The EICESS-92 trial is an example of how collaboration between national clinical study groups is required to run large randomised trials with sufficient power for robust analyses in rare cancers. It is acknowledged that the work has been delayed in its publication but it has been revisited to coincide with a strong current focus and drive for international consensus on the role of surgery and radiotherapy in ES.

The low rates of local recurrence evident with patients undergoing combined modality treatment and the enhanced survival for a cohort of patients, more of whom underwent surgical resection and received radiotherapy, indicates that clinicians should always consider this option. Nevertheless, this must be balanced against the additional late effects, including second malignancies, which are associated with the use of radiotherapy in ES.

## Conclusion

In summary, unexpected differences in survival between cohorts of patients within the same randomised trial have been identified and are national in origin. It appears that less aggressive methods of local control have resulted in a higher rate of local recurrence and this was associated with a higher risk of metastatic disease and subsequent death. These data reinforce the importance of careful planning of treatment for local tumour control in ES and that radiotherapy alone should be discouraged when surgical resection can be undertaken. International clinical trials may offer opportunities to explore the impact of different treatment approaches. As a consequence of the results from this trial, the UK has initiated a system for centralised national review and guidance on local treatment decision making for ES. This system is currently undergoing evaluation.

## Appendix

See Tables 6, 7, 8, 9, 10, 11 and 12.

**Table 6:** Number of patients and events according to trial group

	Number of patients (percentage)		
	CCLG N = 210	GPOH N = 437	Total
First events (total)	119 (57)	204 (47)	323
Death-treatment related	1 (0.5)	3 (1)	4
Disease progression	3 (1)	12 (3)	15
Unknown cause	3 (1)	1 (0.2)	3
Distant metastases	66 (31)	148 (34)	214
[Distant metastases in those with metastatic disease at baseline]	[25 (12%)]	[57 (13%)]	[82]
Local relapse	29 (14)	13 (3)	42
Local and distant relapse	16 (8)	18 (4)	34
Relapse (unspecified site)	0	4 (1)	4
Second malignancy	2 (1)	5 (1)	7
All deaths	105 (50)	168 (38)	273

As a percentage of the number of patients from either CCLG or GPOH

**Table 7:** Distribution of first events by treatment modality: CCLG patients

	Local treatment modality, N (%)						Total
	Surgery alone N = 70	RT alone N = 81	RT then surgery N = 6	Surgery then RT N = 32	None N = 18	Unknown N = 3	
No event	41 (59)	31 (38)	2 (33)	14 (44)	0	2 (67)	90
Local recurrence	5 (7.1)	12 (15)	1 (17)	1 (3.1)	10 (56)	0	29
Distant recurrence	18 (26)	28 (35)	1 (17)	13 (41)	5 (28)	1 (33)	66
Local and distant	3 (4.3)	6 (7.4)	2 (33)	3 (9.4)	2 (11)	0	13
Relapse-unspecified	0	0	0	0	0	0	0
Second malignancy	1 (1.4)	1 (1.2)	0	0	0	0	2
Death—no relapse	2 (2.9)	3 (3.7)	0	1 (3.1)	1 (5.6)	0	7
All deaths	24 (34)	45 (56)	4 (67)	15 (47)	16 (89)	1 (33)	105

*RT* radiotherapy

**Table 8:** Distribution of first events by treatment modality: GPOH patients

	Local treatment modality, N (%)						Total
	Surgery alone N = 71	RT alone N = 85	RT then surgery N = 195	Surgery then RT N = 60	None N = 7	Unknown N = 19	
No event	46 (65)	30 (35)	106 (54)	38 (63)	1 (14)	12 (63)	233
Local recurrence	3 (4.2)	3 (3.5)	3 (1.5)	4 (6.7)	0	0	13
Distant recurrence	19 (27)	36 (42)	71 (36)	16 (27)	2 (29)	4 (21)	148
Local and distant	2 (2.8)	10 (12)	4 (2.0)	1 (1.7)	0	1 (5.3)	18
Relapse-unspecified	0	1 (1.2)	2 (1.0)	0	0	1 (5.3)	4
Second malignancy	1 (1.4)	2 (2.4)	2 (1.0)	0	0	0	5
Death—no relapse	0	3 (3.5)	7 (3.6)	1 (1.7)	4 (57)	1 (5.3)	16
All deaths	18 (25)	47 (55)	75 (38)	17 (28)	6 (86)	5 (26)	158

To one decimal place if < 10%

*RT* radiotherapy

**Table 9:** Hazard ratios for comparing CCLG and GPOH patients

	EFS		OS	
	HR (95% CI)	p value	HR (95% CI)	p value
All patients				
Unadjusted	1.42 (1.13–1.77)	0.002	1.45 (1.14–1.86)	0.003
Unadjusted HR, in localised disease only	1.47 (1.11–1.96)	0.007	1.52 (1.11–2.07)	0.009
Adjusted for risk group and trial treatment	1.43 (1.14–1.79)	0.002	1.49 (1.17–1.91)	0.001
Adjusted for each of the following factors separately^a^				
Age	1.45 (1.15–1.81)	0.001	1.47 (1.15–1.88)	0.002
Metastatic disease	1.38 (1.10–1.73)	0.005	1.42 (1.11–1.81)	0.005
Primary site	1.48 (1.18–1.86)	<0.001	1.52 (1.19–1.95)	0.001
Histology	1.41 (1.12–1.77)	0.004	1.44 (1.12–1.85)	0.004
Local treatment modality^b^	1.45 (1.12–1.89)	0.006	1.30 (0.98–1.72)	0.07
Adjusted for age, metastatic disease, primary site, histology and local treatment^a^	1.44 (1.10–1.89)	0.009	1.30 (0.97–1.74)	0.08
Adjusted HR, in localised disease only	1.48 (1.05–2.09)	0.026	1.29 (0.88–1.89)	0.19
Only patients who had local therapy; excluding progressive disease (n = 25) and where it was not known whether local therapy was given or not n = 22)				
Unadjusted	1.22 (0.96–1.55)	0.11	1.28 (0.99–1.67)	0.06
Adjusted for type of local treatment^c^	1.14 (0.87–1.51)	0.34	1.25 (0.92–1.68)	0.15
Adjusted for time between the start of chemotherapy and starting local treatment	1.12 (0.87–1.44)	0.37	1.18 (0.90–1.55)	0.22
Adjusted for age, metastatic disease, primary site, histology, local treatment, and time between the start of chemotherapy and starting local treatment	1.13 (0.84–1.50)	0.42	1.25 (0.91–1.71)	0.17

Hazard ratios greater than 1 indicate that CCLG patients had a higher risk of having an event or dying compared to GPOH patients

*EFS* event-free survival; *OS* overall survival

^a^Using Cox regression modelling (age as a continuous variable). Missing data for the other variables were included as a separate category, but excluding these from the analyses did not materially change the hazard ratio estimates in the table

^b^Includes categories for no local therapy and missing data

^c^Surgery alone, radiotherapy alone, surgery then radiotherapy, radiotherapy then surgery

**Table 10:** Hazard ratios (CCLG vs. GPOH) according to local treatment modality, among patients with localised disease only

	Local treatment modality			Subdivision of RT and surgery group	
	Radiotherapy (RT) alone N = 108	Surgery alone N = 119	RT and surgery N = 221	RT then surgery N = 152	Surgery then RT N = 69
No. events					
EFS	62	42	83	61	22
OS	53	33	66	49	17
Unadjusted					
EFS	0.89 (0.54–1.46)	1.44 (0.78–2.64)	1.66 (0.94–2.96)	2.44 (0.76–7.81)	2.36 (1.02–5.45)
OS	0.99 (0.56–1.64)	1.74 (0.86–3.51)	1.60 (0.84–3.06)	2.70 (0.84–8.73)	2.03 (0.78–5.28)
Adjusted for age, primary site and histology					
EFS	0.98 (0.58–1.66)	1.51 (0.78–2.94)	1.65 (0.91–2.99)	2.39 (0.71–8.05)	2.12 (0.83–5.40)
OS	1.10 (0.62–1.95)	1.94 (0.89–4.25)	1.50 (0.77–2.93)	3.10 (0.90–10.70)	1.74 (0.60–5.08)
Adjusted for age, primary site, histology and time between the start of chemotherapy and starting local treatment					
EFS	0.95 (0.54–1.67)	1.48 (0.76–2.89)	1.48 (0.81–2.69)	1.69 (0.50–5.68)	2.10 (0.82–5.41)
OS	1.03 (0.55–1.90)	1.92 (0.87–4.24)	1.39 (0.71–2.74)	2.49 (0.72–8.58)	1.74 (0.59–5.10)

Hazard ratios greater than 1 indicate that CCLG patients had a higher risk of having an event or dying compared to GPOH patients

Based on data excluding patients with unknown primary site because there were so few

*EFS* event-free survival; *OS* overall survival

**Table 11:** Hazard ratios (CCLG vs. GPOH) according to local treatment modality, among patients with metastatic disease only

	Local treatment modality		
	Radiotherapy (RT) alone N = 56	Surgery alone N = 19	RT and surgery N = 66
No. events			
EFS	43	11	48
OS	39	8	43
Unadjusted			
EFS	0.79 (0.43–1.44)	0.79 (0.24–2.61)	2.01 (0.89–4.54)
OS	0.92 (0.49–1.74)	0.71 (0.18–2.85)	1.98 (0.88–4.47)
Adjusted for age, primary site, histology and time between the start of chemotherapy and starting local treatment			
EFS	0.81 (0.44–1.67)	Too few patients to allow for other factors reliably	2.51 (0.89–7.06)
OS	1.01 (0.50–2.01)		2.54 (0.90–7.20)

Hazard ratios greater than 1 indicate that CCLG patients had a higher risk of having an event or dying compared to GPOH patients

Based on data excluding patients with unknown primary site because there were so few

*EFS* event-free survival; *OS* overall survival

**Table 12:** Hazard ratios for comparing CCLG and GPOH patients, after examining local treatment and time to local treatment

	EFS		OS	
	HR (95% CI)	p value	HR (95% CI)	p value
All patients				
Unadjusted	1.22 (0.96–1.56)	0.10	1.29 (0.99–1.68)	0.055
Adjusted for local treatment^a^	1.14 (0.87–1.51)	0.34	1.25 (0.93–1.68)	0.15
Adjusted for between the start of chemotherapy and starting local treatment	1.13 (0.88–1.45)	0.34	1.19 (0.91–1.56)	0.20
Adjusted for both local treatment and timing	1.11 (0.84–1.47)	0.46	1.21 (0.90–1.65)	0.21
Localised disease only				
Unadjusted	1.31 (0.97–1.76)	0.08	1.39 (1.00–1.93)	0.048
Adjusted for local treatment^a^	1.22 (0.87–1.72)	0.25	1.30 (0.89–1.90)	0.17
Adjusted for between the start of chemotherapy and starting local treatment	1.18 (0.87–1.60)	0.30	1.24 (0.88–1.74)	0.21
Adjusted for local treatment and the time between the start of chemotherapy and starting local treatment	1.14 (0.81–1.62)	0.45	1.20 (0.82–1.77)	0.35
Metastatic disease only				
Unadjusted	0.98 (0.65–1.48)	0.93	1.01 (0.66–1.57)	0.94
Adjusted for local treatment^a^	0.98 (0.61–1.56)	0.93	1.11 (0.68–1.81)	0.69
Adjusted for between the start of chemotherapy and starting local treatment	0.95 (0.62–1.45)	0.81	1.00 (0.64–1.57)	0.98
Adjusted for both local treatment and timing	0.99 (0.61–1.60)	0.97	1.14 (0.69–1.89)	0.60

Type of local treatment and time to local treatment seem to be independent factors. In the Cox regression which contains both of them, the p values for each variable are: All patients EFS: local treatment (p < 0.0001); time to local treatment (p < 0.001)

All patients OS: local treatment (p < 0.0001); time to local treatment (p < 0.001)

Patients with localised disease only, EFS: local treatment (p = 0.002); time to local treatment (p = 0.002)

Patients with localised disease only OS: local treatment (p = 0.002; time to local treatment (p = 0.004)

^a^Surgery alone, radiotherapy alone, surgery then radiotherapy, radiotherapy then surgery
